# Spatial and temporal patterns in the sex ratio of American lobsters (*Homarus americanus*) in southwestern Nova Scotia, Canada

**DOI:** 10.1038/s41598-021-03233-8

**Published:** 2021-12-16

**Authors:** S. Koepper, C. W. Revie, H. Stryhn, K. F. Clark, S. Scott-Tibbetts, K. K. Thakur

**Affiliations:** 1 Department of Health Management, Atlantic Veterinary College, University of Prince Edward Island, Charlottetown, C1A 4P3 PE Canada; 2 Department of Computer and Information Sciences, University of Strathclyde, Glasgow, G1 1XQ UK; 3 Department of Animal Sciences and Aquaculture, Faculty of Agriculture, Dalhousie University, Truro, NS B2N 5E3 Canada; 4 Fishermen and Scientists Research Society, Halifax, NS B3M 4H4 Canada

**Keywords:** Population dynamics, Ocean sciences

## Abstract

An approximate 1:1 sex ratio of American lobsters can be skewed due to environmental factors or fisheries management. Substantial skewness can impact mating behaviour and lower reproduction which could have far-reaching ecological and economic consequences. The aim was to investigate the sex ratio patterns of lobsters in two lobster fishing areas (LFAs) in southwestern Nova Scotia, Canada and identify factors associated with skewed sex ratios. This study analyzed biological data from more than 270,000 lobsters sampled over ten years (2010–2019) by the Fishermen and Scientists Research Society. A mixed effect logistic regression model evaluated the effect of spatial, temporal and environmental factors as well as size on the sex ratio of lobsters. There were significant temporal patterns in sex ratios that differed by LFA. After the effects of sampling month, year and LFA were accounted for, lower bottom temperature and deeper water depth were associated with a higher prevalence of females, especially in larger lobsters. We present the first long term analyses of sex ratio patterns in *H. americanus* in Atlantic Canada’s most commercially important region for this species and provide evidence that these patterns are influenced by environmental factors and fisheries. In view of future climate change scenarios, monitoring the population dynamics of this iconic fishery species is crucial to ensure sustainable fisheries and healthy lobster stocks.

## Introduction

The American lobster (*Homarus americanus* H. Milne-Edwards, 1837) is not only an important link in the food web of benthic marine communities, it is also the most valuable fisheries product in Atlantic Canada^1,2^. Lobster landings in 2019 made up approximately 20% of the total fisheries catch in Atlantic Canada (103,917 mt / 560,484 mt total), while accounting for almost 50% of its total commercial value ($1.6B CAD / $3.2B CAD total)^2^. Consequently, the lobster fishery is an important economic sector and employer in the Maritime region and relies on a well-managed lobster population. Fisheries regulations such as minimum legal sizes, v-notching (in some management areas) and protection of ovigerous (egg bearing) and larger females have been implemented in Canadian waters to ensure that the American lobster fishery remains sustainable^3^.

American lobsters usually have an approximate 1:1 ratio of males and females and while it is not required to have the same amount of male and female lobsters to uphold a stable population, substantial or long lasting changes in sex ratio can affect mating, reproduction and population dynamics^4–7^. Sperm-limitation, where there are not enough reproductive males to fertilize all the eggs in the population^8,9^, has been reported in female-skewed crustacean populations^6,10–13^. The causes of sex ratio shifts in lobster populations are poorly understood; but they are crucial for any species under a sex-biased harvest, such as lobsters where larger and ovigerous females cannot be fished^5^.

Sex and size specific fisheries regulations lead to an increased removal of larger males. Therefore, larger females should have a lower fishing mortality and remain in the population, resulting in a female skewed sex ratio^3,5^. A male targeted lobster fishery may result in apparent female-biased sex ratio estimates because data (especially large scale) on catch composition are often collected by fisheries surveys due to convenience. Another aspect influencing the observed sex ratio estimates is that male and female lobsters can differ in their catchability. For example, an earlier moult in males leads to an earlier onset of post-moult foraging behaviour, which makes males more likely to encounter traps at that time^5,14^. Other factors also include individual behaviour and motivation to interact with fishing gear which can obscure the interpretability of trap-based sex ratio data^15,16^. It is critical to recognize that short-term population surveys (e.g. single time point samplings) can result in estimates that are biased towards either sex, depending on which is more likely to be captured at that time while long-term fishery monitoring will reveal less confounded sex ratios.

Environmental factors, such as temperature and salinity, have been reported as drivers of short and long term migration patterns in marine crustaceans^4,17,18^. As ectotherms with a thermal window between 0 and 20 °C, lobsters are very sensitive to temperature changes and can thermoregulate behaviourally, i.e. migrate to a more favourable environment for moulting, mating or egg release^16,19–22^. Seasonal migrations of adult lobsters from offshore in the winter to inshore in the summer are well documented; most likely due to seasonal shifts in food abundance and a preference in physical conditions^23^. It is also known that male and female lobsters react differently to physical conditions^24,25^. For example, females generally prefer colder waters more than males and ovigerous specimens migrate between shallow and deep waters to reduce temperature variability for their brood^5,16^. Females are also more likely to avoid lower salinities due to lower osmoregulation capabilities which is believed to be the reason why estuarine waters have been shown to be male dominated^5^. Therefore, it should be assumed that changes in environmental conditions can also affect the sex ratio of lobsters.

Sea temperatures in the western North Atlantic are predicted to rise at higher rates than the global average and reported shelf ocean temperatures in southern Atlantic Canada indicate a steady increase over the last decades^26,27^. Climate change is expected to impact lobsters as increases in benthic marine temperatures over their optimal thermal range can negatively impact growth, recruitment and survival and eventually population dynamics^28,29^.

Our goal was to describe spatio-temporal patterns in the sex ratio and to determine effects of bottom temperature, water depth and lobster life history on sex-based lobster distribution patterns with a mixed effect logistic regression model. To the best of our knowledge, this is the first comprehensive study on sex ratio patterns of lobsters in Atlantic Canada.

## Materials and methods

### Data collection

The Fishermen and Scientists Research Society (FSRS) sampled lobsters in LFA (Lobster Fishing Area) 33 and 34 during the lobster fishing season (November–May) every year as part of the continuously ongoing Lobster Recruitment Index project. The dataset used here contained data from 2010–2019. Lobsters were collected by volunteer fishers using single scientific traps which were smaller than commercially fished traps (101.6 cm × 35.6 cm × 53.3 cm, 2.54 cm mesh size). No escape vents were present in the scientific traps to ensure that a wider size of lobsters was included in the study compared to conventional traps. Soak times for traps and baiting practices were not standardized among the vessels and over the study period. Common bait consisted of mackerel, herring and redfish but this information and the amount of bait used per trap was not available for our study. While soak times had no significant effect on sex ratios, any observed differences in sex ratios were after accounting for different soak times. Data on lobster sex (determined by examining the first set of pleopods), size (15 size bins), fishing location (GPS coordinates) and water depth were recorded. Bottom temperature was measured by VEMCO loggers attached to the traps.

### Descriptive statistics

All data were analyzed using Stata (v. 17; StataCorp, 2021: https://stata.com) and descriptive analyses were performed to summarize the data. Prior to analysis, the categorical variable lobster size referred from now on as carapace length (CL) was regrouped from 15 into four new categories (1: < 60.9 mm CL, N = 31,631; 2: 61–80.9 mm CL, N = 145,221; 3: 81–100.9 mm CL, N = 86,006; 4: > 101 mm CL, N = 9,715, legal size = 82.5 mm CL) because of high variation in the number of observations in the original size bins. The raw data was visualized using histograms and scatter plots for continuous variables and by frequency tables for categorical factors to assess their distribution and variability. Histograms were also used to explore whether the sex ratio was biased by lobster size distribution, if there were larger males, and smaller females. Sampling locations were mapped using QGIS (v. 3.18; QGIS Geographic Information System, 2021; http://www.qgis.org/)^30^ and distance to shore was calculated for each location by QGIS’s NNJoin panel (v. 3.1.3; https://plugins.qgis.org/plugins/NNJoin/)^31^. To visualize spatial effects on the sex ratio, the average proportion of males for each sampling location was calculated, categorized (< 20%, 20–40%, 40–60%, 60–80%, > 80% males) and color coded.

### Selection of model outcome

Instead of using a continuous outcome (proportion of male or female lobsters captured within a trap or sampling event) and a linear regression model, it was decided to use a logistic regression model in which lobster sex was represented as a binary outcome (male = 1, female = 0)^32^. The linear outcome would be based on proportion of males in each sampling event. In similar studies this resulted in reliable predictions from continuous models as there were a sufficient numbers of observations per sampling event^33^. Here however, on average only 12 lobsters (range 1–251) were sampled per sampling event, which led to the proportion of males value being close to 0 and 1 in many cases. A linear model violated the assumptions of normally distributed residuals and homoscedasticity. Hence, logistic regression was assumed to fit the data more effectively and the probability of sampling a male lobster was modeled.

### Mixed effect logistic regression model

Our mixed effect logistic regression model included the sampling event, i.e. lobsters fished on the same day by the same vessel, as a random effect. The unconditional associations between the outcome variable and each predictor were checked respectively in univariate regression with a liberal p-value of 0.2 (using Wald-test).

Pearson correlation coefficients for parametric data and Spearman rank correlation coefficients for non-parametric data were calculated to assess collinearity, especially between the variables bottom temperature, water depth and distance to shore as they are physically highly dependent on one another. A causal diagram showing the effects of factors on lobster sex ratio patterns is provided in the supplement material (Fig. S1). Confounding between main factors and model outcome was assessed after Dohoo et al.^34^. Due to high collinearity between water depth and distance to shore (r = 0.74, p < 0.001), distance to shore was excluded from model building, though it was highly significant in univariate regression. Water depth and bottom temperature were also correlated but excluding water depth from the model confounded the coefficient for bottom temperature by more than 20% and therefore it was decided to include both factors in the model.

Two-way interactions were modeled between the spatio-temporal factors (year-LFA, month-LFA) as well as between the environmental factors and carapace length (temperature-size, depth-size). Significant interactions were kept in the model if they improved model fit by 5% based on AIC. Including two possible three-way interactions (year-month-LFA, depth-temperature-size) did not improve the model performance compared to the model with four two-way interactions and therefore the final regression model included only two-way interactions (see supplementary Table S1 for AIC data).

Manual forward selection was performed to build the final model on which regression diagnostics were performed: Pearson residuals were checked based on the covariate patterns (unique combinations of values of predictor variables), and important or influential observations were assessed by calculating leverage, delta-chi^2^ and delta-beta values for each observation. Clustering of observations within sampling events was assessed by computing the intraclass correlation coefficients (ICC). Unless specifically stated otherwise, a significance level of 0.05 was used for all statistical analyses. The resulting final mixed logistic regression model is listed below (*β*_0_ = model intercept, *β*_*1-4*_ = slope, *u* = random effect, *ε* = error):

1 $$\begin{gathered} Logit\left( {{\text{p}}_{{{\text{male}}}} } \right) \, = {\beta}_{0} + {\beta}_{{1}} \left( {{\text{Month}}*{\text{LFA}}} \right) \, + {\beta}_{{2}} \left( {{\text{Year}}*{\text{LFA}}} \right) \hfill \\ + {\beta}_{{3}} \left( {{\text{Temperature}}*{\text{size}}} \right) \, + {\beta}_{{4}} \left( {{\text{Depth}}*{\text{size}}} \right) \, + u_{{\text{Sampling event}}} + \varepsilon \hfill \\ \end{gathered}$$

### Model predictions for relevant environmental scenarios

To visualize the individual effects of sampling month, sampling year and LFA on the sex ratio patterns, all other factors were fixed to environmentally meaningful values in two constructed scenarios. The first modelled scenario estimated the sex ratio for a lobster population in shallow, warm waters (5 m water depth, 10 °C bottom temperature), while the second scenario estimated the sex ratio in deep, cold waters (50 m water depth, 3 °C bottom temperature). In both scenarios, the lobster size was fixed to size category three (81–100.9 mm CL), as these lobsters are above minimum legal size and therefore the most relevant to the fisheries.

## Results

### Descriptive statistics

**Figure 1:**
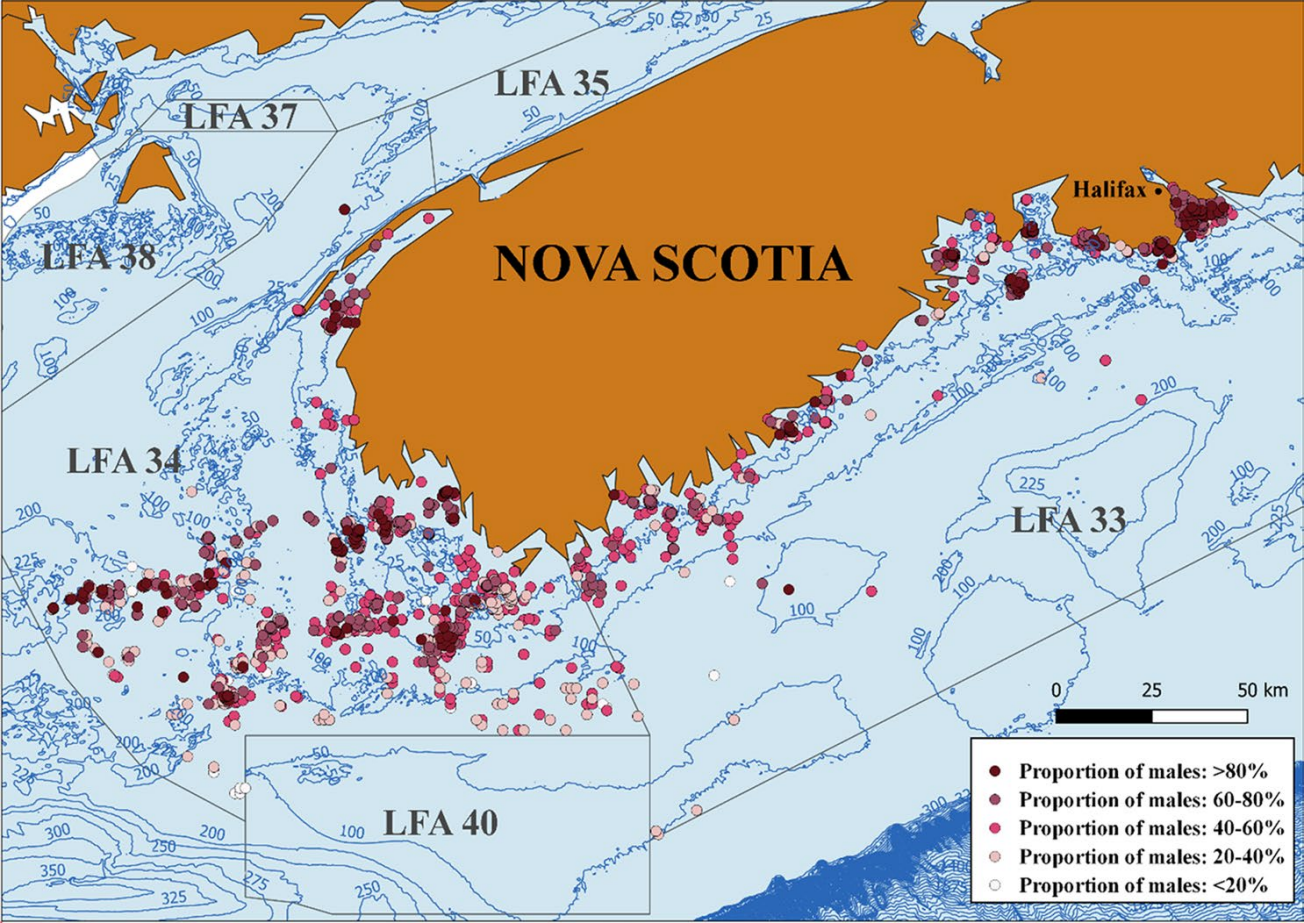
Map of sample area with observed sex ratio (proportion of males) for each lobster fishing location over the study period. Map was created with QGIS (v 3.18; https://www.qgis.org/) and LFA profiles were obtained from the ALMQ project (Atlantic Lobster Molt and Quality; www.fsrsns.ca). Contours depict water depths in meters.

The FSRS dataset contained observations from 272,573 lobsters sampled from 2010 to 2019 in 3330 locations and 22,973 sampling events from lobster fishing areas (LFA) 33 and 34. The overall proportion of males was slightly higher than the proportion of females (0.521, 95% CI 0.519–0.524). Descriptive statistics for the continuous factors bottom temperature, water depth, soak times and distance to shore are summarized in Table 1 and the annual frequency of samples and proportions of males in the two LFAs are presented as supplementary information in Table S2. Figure 1 illustrates the observed sex ratio in each sampled location over the sampling period.

**Table 1:** Summary statistics of continuous factors bottom temperature, water depth, distance to shore and soak times by LFA (calculated on sampling event level). The 95% confidence intervals for the means are shown in brackets.

		LFA					
		33			34		
	N	Mean	Min	Max	Mean	Min	Max
Temperature (°C)	249,961	5.76 (5.71, 5.81)	− 1.34	12.00	6.63 (6.57, 6.68)	− 0.85	12.27
Depth (m)	271,770	22.14 (21.65, 22.62)	1.83	170.09	30.54 (29.94, 31.13)	3.66	210.32
Distance (km)	272,573	4.77 (4.63, 4.91)	0.01	101.41	15.24 (14.82, 15.67)	0.01	121.08
Soak times (days)	272,573	2.24 (2.23, 2.25)	1	50	2.75 (2.73, 2.77)	0.75	50

There was a weak positive correlation between bottom temperature and water depth (Spearman rank correlation coefficient r_s_ = 0.12, N = 249,162, p < 0.001) associated with lobsters caught in the study. This may be due to higher seasonal variability in shallow (0–25 m: N = 119,260, SD = 2.14) than in deeper waters (25–50 m: N = 19,180, SD = 1.93).

**Figure 2:**
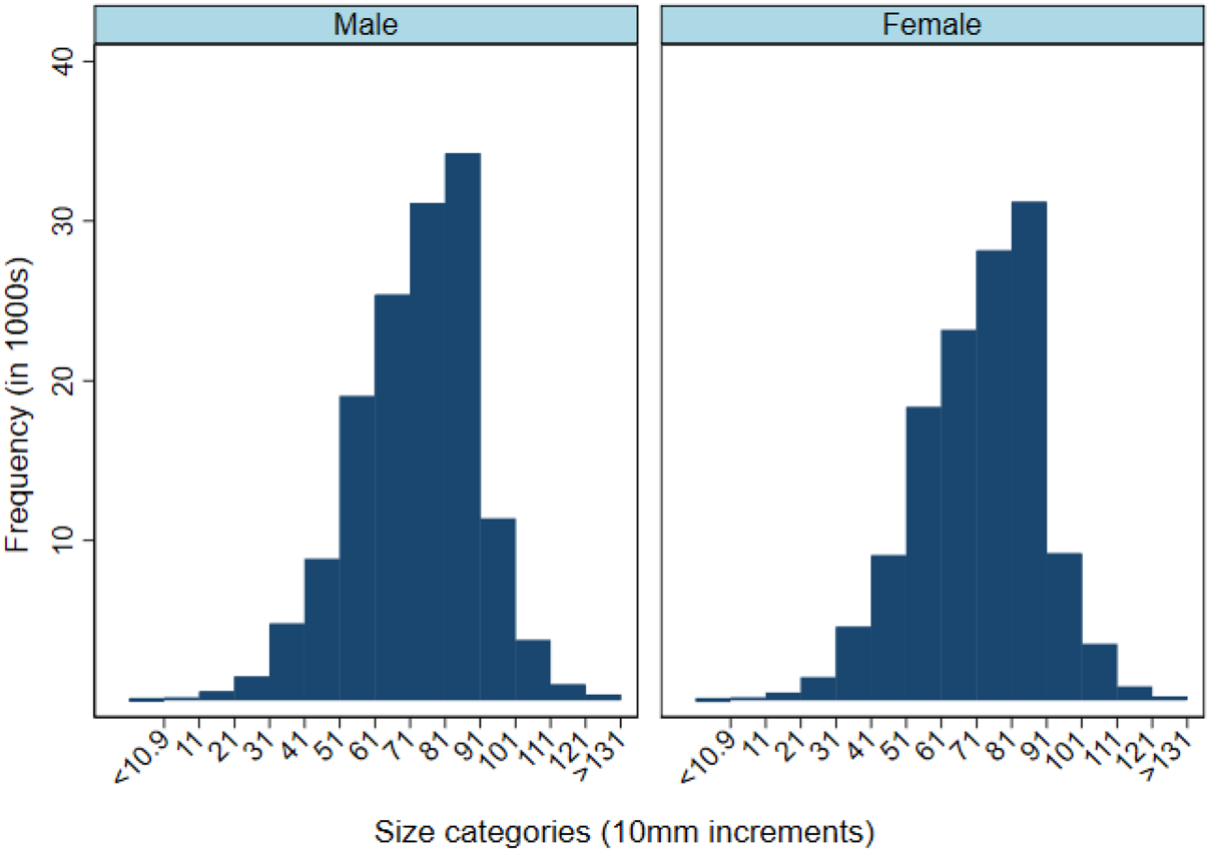
Size distributions of male and female lobsters from 2010–2019 in southwestern Nova Scotia, Canada.

Male and female lobsters in the catch had a similar, unimodal size distribution with a peak at 71–80.9 mm CL, just below the minimum legal size. For both sexes, sizes below 21 mm CL and above 121 mm CL were rare (Fig. 2). The smallest lobsters cannot be sampled with traps as they can escape through holes in the traps and are not rare in the population, while the largest lobsters are presumably rare in the population due to fisheries removal.

### Mixed effect logistic regression

Distance to shore had a significant effect on the sex ratio when included in the mixed effect logistic regression model as the only predictor (OR = 0.99, p < 0.001). Figure 1 illustrates the proportion of males in each sampling location over the study period. Despite clustering in densely sampled areas, southern offshore locations had a lower prevalence of males than locations closer to the coast. According to the univariate logistic model, for every kilometer increase in distance to shore the odds of being male decreased by 1%. However, due to collinearity (r = 0.74, p < 0.001) with water depth this predictor was excluded from the final model.

Due to missing temperature values for some samples, the final mixed logistic regression model included 249,162 observations and found significant interactions between month and LFA (p < 0.001), year and LFA (p < 0.001), bottom temperature and size (p < 0.001) and water depth and size (p < 0.001). In this mixed logistic regression, the intraclass correlation coefficient (ICC) within sampling events was 0.008, indicating no strong clustering. This indicates that lobsters sampled by the same vessel on the same day did not resemble each other in terms of their sex ratios, once the fixed effect had been accounted for.

### Monthly and yearly sex ratio patterns

**Figure 3:**
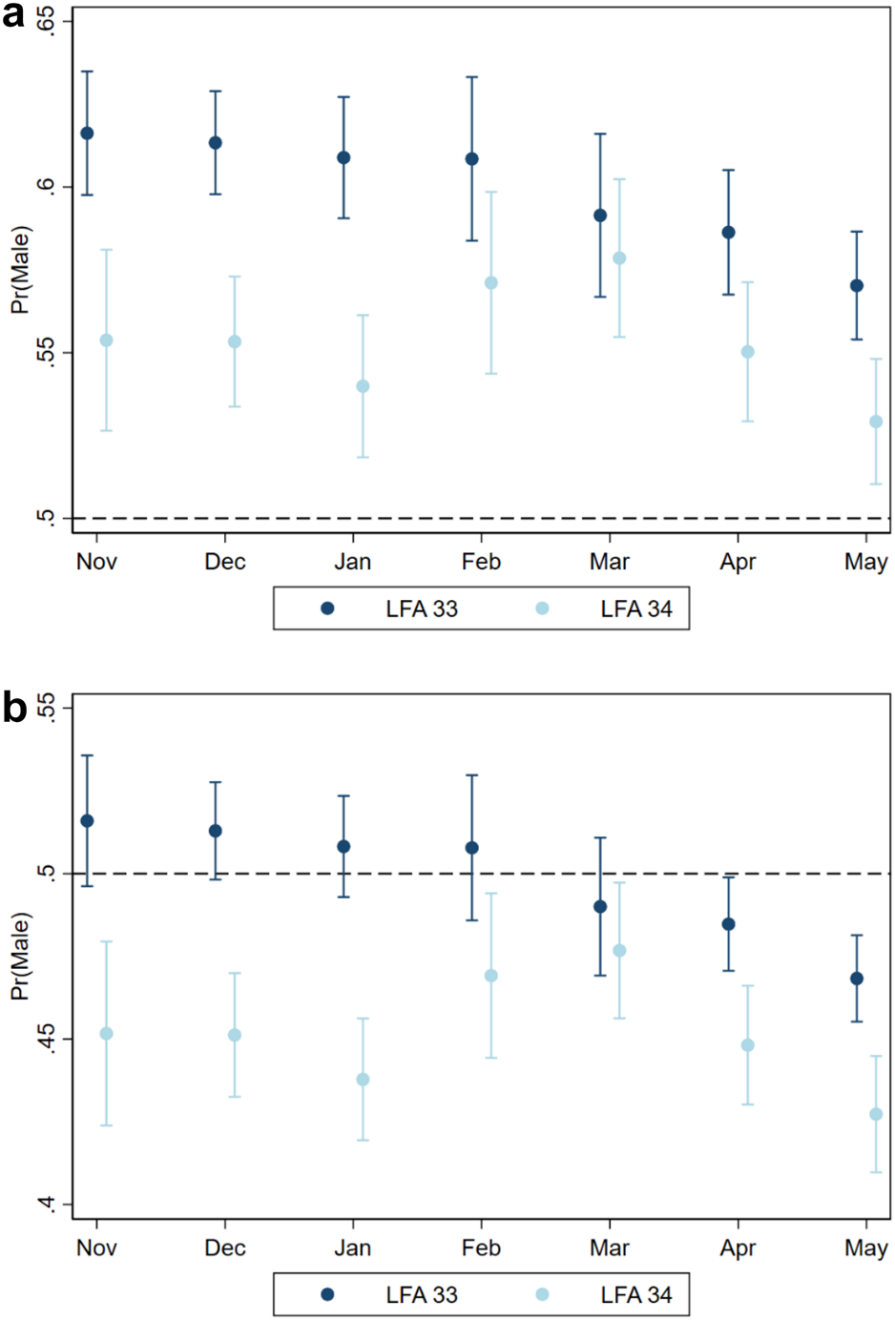
Estimated probabilities (with 95% confidence intervals) of sampling male lobsters per LFA and sampling month from a mixed effect logistic regression (N = 249,961) with year, temperature, size and water depth as fixed effects and sampling event as a random effect. (**a**) Predictors in model fixed to a shallow, warm water scenario (10 °C, 5 m depth for lobsters from 81–100.9 mm CL sampled in the year 2019). (**b**) Predictors in model fixed to a deep, cold water scenario (3 °C, 50 m depth for lobsters from 81–100.9 mm CL sampled in the year 2019). The dotted line along the Y axes represents a 1:1 sex ratio. Note that Y axis scales differ to accommodate the range of estimates.

The probability of sampling males differed among sampling months and depended on the LFA (Fig. 3). Notably, the two areas differed in male sampling probability at the beginning of the lobster fishing season (Nov–Jan), while they were more similar from February to May. In LFA 33, the probability of sampling males declined towards the end of the fishing season (March–May), while in LFA 34 the probability of males was not lower at the end of the fishing season (May) compared to the start (November). The output from the two modelled scenarios, low water depth and higher temperature vs. high water depth and lower temperature (Fig. 3a,b), showed that the probability of sampling males was consistently higher in warm and shallow waters as compared to deeper, cold waters.

**Figure 4:**
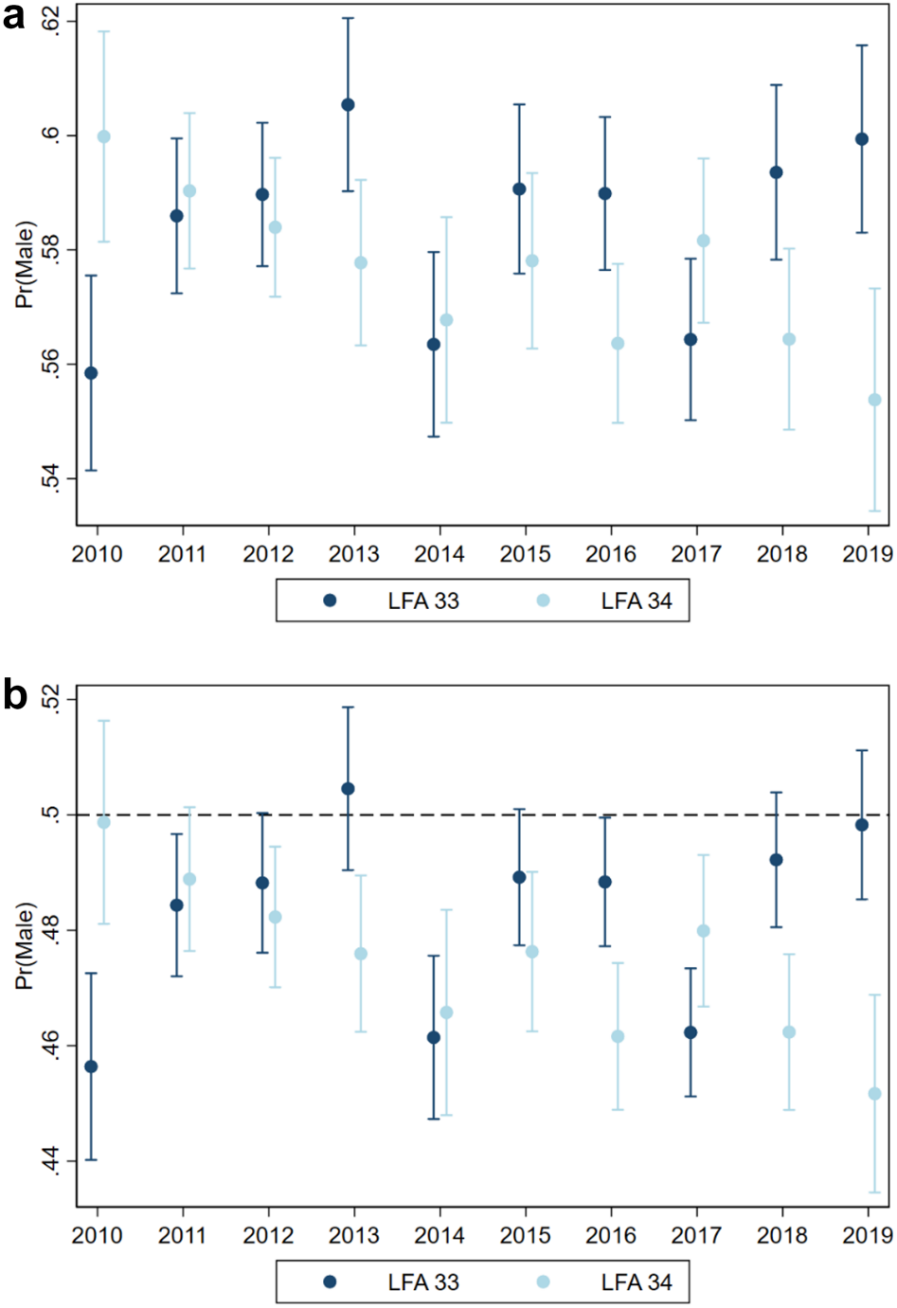
Estimated probabilities (with 95% confidence intervals) of sampling male lobsters per LFA and sampling year from a mixed effect logistic regression (N = 249,961) with month, temperature, size and water depth as fixed effects and sampling event as a random effect. (**a**) Predictors in model fixed to a shallow, warm water scenario (10 °C, 5 m depth for lobsters from 81–100.9 mm CL sampled in the month of May). (**b**) Predictors in model fixed to a deep, cold water scenario (3 °C, 50 m depth for lobsters from 81–100.9 mm CL sampled in the month of May). The dotted line along the Y axes represents a 1:1 sex ratio. Note that Y axis scales differ to accommodate the range of estimates.

The temporal effect of sampling years on the sex ratio of lobsters also depended on the LFAs and is presented in Fig. 4. Over the ten-year study period (2010–2019), the probability of sampling males decreased in LFA 34, while increased in LFA 33. These opposing trends are especially pronounced from 2010–2013 and from 2017–2019. In LFA 33, the probability of sampling males decreased sharply from 2013 to 2014 from 0.56 to 0.51. Similar to the monthly sex ratio patterns for the two modelled scenarios, the probability of sampling males was higher in the warm, shallow water scenario compared to the cold, deep water scenario (Fig. 4a,b).

### Effects of environmental factors and lobster size on sex ratio patterns

**Figure 5:**
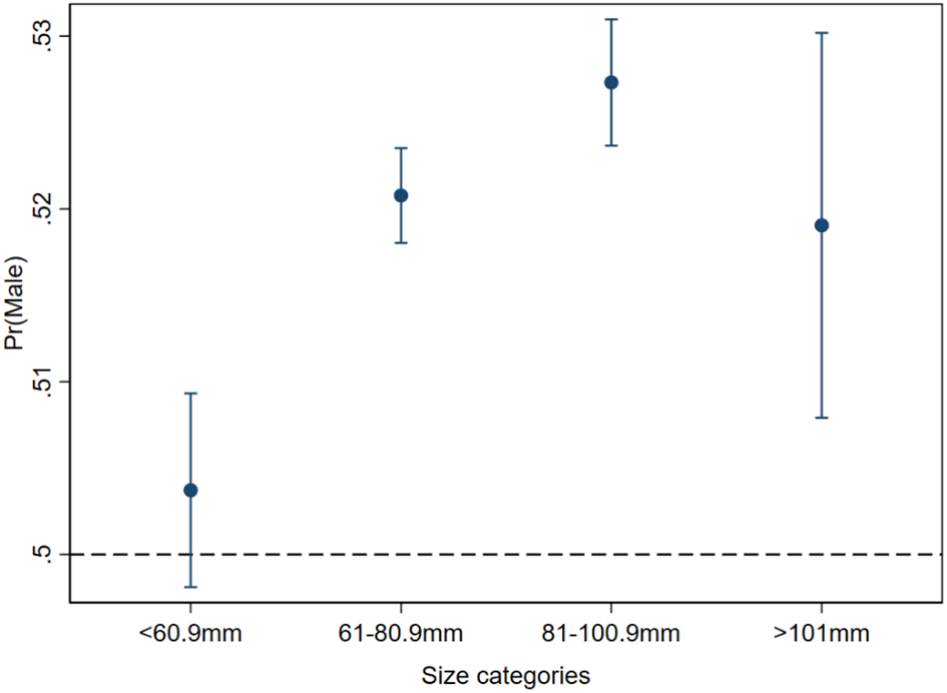
Estimated effects of carapace length on the probability of sampling males from a mixed logistic regression with month-LFA, year-LFA, bottom temperature, water depth and carapace length as fixed and sampling event as random effect.

When only the single effect of carapace length was evaluated, the mixed effect logistic regression output of sex ratio in the four size classes indicated that the probability of sampling males increased with size but levelled off for lobsters in the largest size class (> 101 mm CL, Fig. 5). Except for lobsters in the smallest size class (< 60.9 mm CL) the model predicted a male-skewed sex ratio.

**Figure 6:**
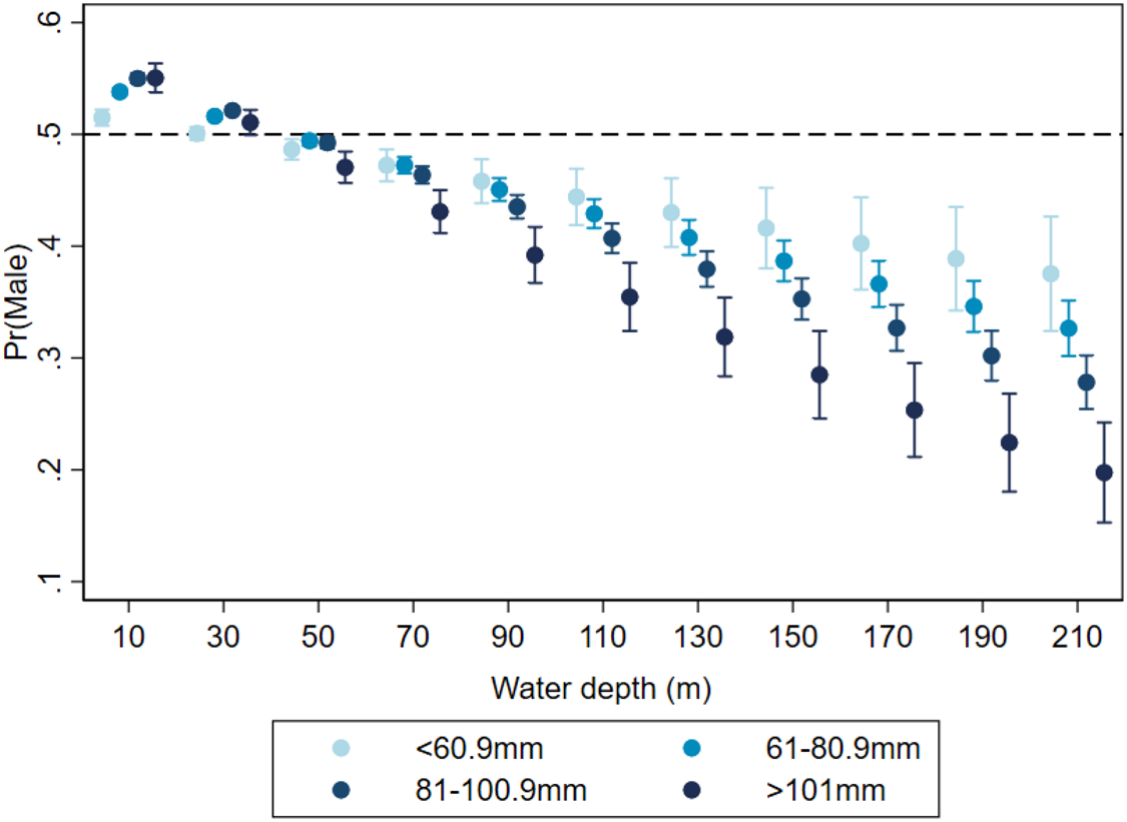
Estimated probabilities of sampling male lobsters by different size categories over increasing water depth. Obtained from a mixed effect logistic regression (N = 249,961) with month-LFA, year-LFA, water temperature-size and water depth-size as fixed effects and sampling events as random effect.

The final mixed logistic regression model showed that the impact of water depth on the sex ratio depended on lobster size. In general, the probability of sampling males decreased in deeper waters, where the sex proportion was skewed towards female lobsters at depths below 40 m. The interaction effect between water depth and size showed that the probability of sampling males decreased the most in larger lobsters (> 101 mm CL, Fig. 6) from 55% at 10 m water depth to 23% at 190 m water depth. This means that it was four times more likely to catch larger (> 101 mm CL) females than males in very deep waters. At the mean sampling depth of 25 m, lobsters in the smallest size class had a closer to 50% (51%) male sampling probability than lobsters in the other three size classes.

**Figure 7:**
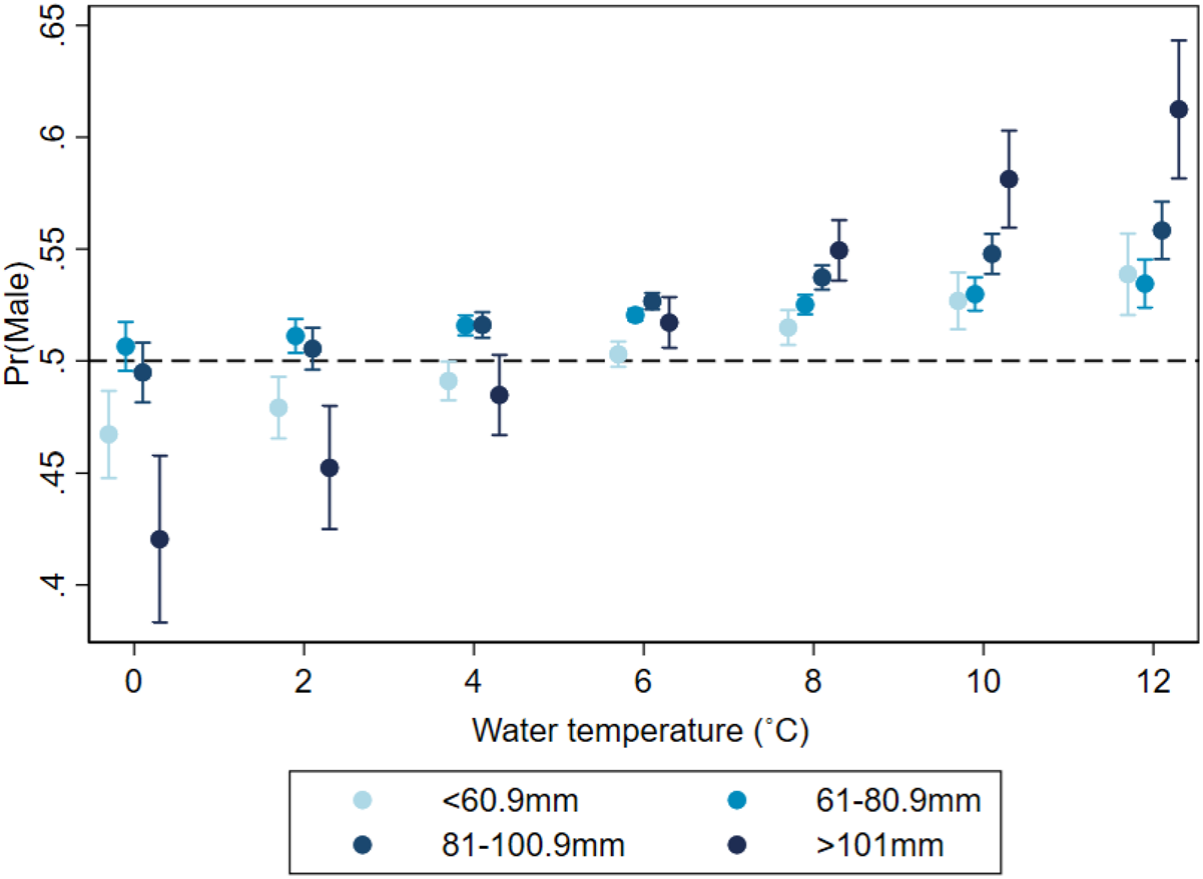
Estimated probabilities of sampling male lobsters by different size categories over increasing water temperature. Mixed effect logistic regression with month-LFA, year-LFA, water temperature-size and water depth-size as fixed effects and sampling events as random effects.

There was also a strong association between sea bottom temperature and sex ratio which also depended on lobster size. In general, the higher the bottom temperature, the higher was the probability of sampling male lobsters. As for water depth, bottom temperature seemed to have the strongest effect in the largest size class (> 101 mm CL) where the probability of sampling males increased from 42% at 0 °C to 61% at 12 °C bottom temperature. For lobsters in size classes one and three (> 60.9 mm cL, 81–100.0 mm CL) the probability of sampling males increased with increasing bottom temperature from 47 to 54% and from 50 to 56% respectively. Lobsters between 61–80.9 mm CL showed the least increase in male sampling probability from 51 to 53% (Fig. 7).

## Discussion

This study investigated the sex ratio patterns in lobsters (*Homarus americanus*) sampled from 2010 to 2019 in southwestern Nova Scotia, Canada. The analyses included data from the two commercially most important LFAs (33, 34) in Canada. The presented results are therefore highly relevant to the Canadian lobster fisheries and its management. We detected differences in the sex ratio due to sampling time, LFA, bottom temperature, water depth and lobster size using a mixed effect logistic regression model.

The temporal effects of sampling year and sampling month both depended on the geographical area (LFAs). Although close in proximity, harvesting strategies differ between LFAs 33 and 34. In LFA 34, the lobster fisheries expanded offshore in the 1980s (up to 90 km away from the coast), while in LFA 33 most of the catch is harvested within 15 km of the coast^35–37^. This differential allocation of fishing grounds was also reflected in our data. The output of the mixed effect logistic regression model accounted for water depth, and indirectly also distance to shore as these two variables showed a strong positive correlation in the descriptive statistics. The spatial differences between the two LFAs observed in the monthly sex ratio patterns therefore may have derived from other unmeasured factors. These two LFAs experience different oceanographical conditions as LFA 34 is adjacent to the Gulf of Maine while LFA 33 is situated on the Atlantic Ocean^38,39^. Different ocean currents, their velocity and direction, ocean productivity or the benthic habitat can influence the salinity, abundance of predators as well as larval recruitment and settlement which are important factors for lobster distribution^17,39–42^. However, we did not have access to these data to account for any effects on sex ratio in our study.

The variability in sex ratio over the course of the calendar year coincides with findings of other studies in *H. americanus*^4,5,17^ and other commercially fished crustaceans^43–45^. This finding can be partly explained by the seasonality of lobster fisheries which is mandated by catch regulations^6^. Our data show a decrease in the probability of male lobster towards the end of the fishing season from March to May (in LFA 33). High fishing pressure during that time could cause a systematic depletion of males and skew the sex ratio towards females. Naturally, seasonality plays an important role in the migration of *H. americanus*, with the direction and magnitude being influenced by the environmental and reproductive preferences of males and females^23,46–48^. We explored likely scenarios by fixing bottom temperature and water depth to shallow, warm water and deep, cold water conditions. This accounts for the most meaningful parameters for lobster migration^16^ and means that the significant effect of the calendar months identified illustrates the likely impact of the fishing pressure on the lobster population.

Even with fixed bottom temperature and water depth metrics, our model predicted interannual changes in the sex ratio and differences between the two LFAs. Despite having the same catch regulations, male probability decreased in LFA 34 and increased in LFA 33^3^—a trend in both regions that cannot solely be attributed to random variation. Higher catch rates and lobster landings in LFA 34 (375–400 traps per fisher and 16,000 mt landed in LFA 34 vs. 250 traps and 8155 mt landed in LFA 33) indicate a higher fishing pressure which in turn could explain the increase in probability of females in this LFA^3,5^.

Our results demonstrate differences in sex ratio based on the lobster fishing areas and suggest a significant influence of fisheries on lobster population dynamics. This could lead to management issues if catch regulations are kept the same in both areas but the lobster population dynamics and distributions change regionally^17^. For example, a decrease in the proportion of males in the population, a trend that we predicted for LFA 33 over the study period, could have impacts on the reproductive output of this stock in the future and may require adjustment of minimum legal sizes (to ensure sufficient large/reproductive lobsters in the population) or fishing season (to allow enough time for reproduction).

Both bottom temperature and water depth were significant factors in estimating the sex ratio of lobsters. Lobster movements are dictated by water temperature and depth, which affects their growth, reproduction, and survival^21,49–51^ and have been reported to lead to skewed sex ratios in previous case studies, where female skewed sex ratios were observed in deep and cold waters^5^. The same trend was also seen in the present study when the inter-monthly and inter-annual sex ratio was modelled under warm/shallow and cold/deep water scenarios. The predicted sex ratios were noticeably skewed towards females in colder and deeper waters. However, in previous studies it was not obvious whether water depth, water temperature or other factors (e.g. currents, salinity) played a more important role for females to choose these areas^5,17^. The predictions from our logistic regression model showed that male probability decreased at a higher rate with increasing water depth than it increased with increasing bottom temperature. It also predicted a female skewed sex ratio in deeper waters and estimated that this effect of water depth on male probability increased from smaller to larger lobsters. Since water depth and temperature are correlated, it also predicted a male skewed sex ratio in warmer waters and estimated that this temperature effect was most impactful in larger lobsters. In tagging studies, ovigerous females have been reported to prefer stable temperatures in deeper waters and that lobster migrations do not always aim for higher or lower temperatures but to avoid high temperature fluctuations^16,23^. This preference of female lobsters for deeper areas could explain why water depth seems to be more influential in our model than water temperature.

Depending on environmental predictors, lobster size significantly influences sex ratio patterns in our model which could be explained by the different growth rate of males and females. Males can maximize growth in warmer water whereas females need to spend time at colder temperatures below 5–8 °C as those are required for ovarian development to ensure reproductive success^48^. Females also need to spend energy and resources on egg development that cannot be invested in growth^52^. However, our results indicate that the size distributions of male and female lobsters were similar overall.

The mixed effect logistic regression model shows a higher probability of males in size classes just above and below the minimum legal size (61–80.9 mm CL and 81–100.9 mm CL). Other studies have also reported a higher skewness in sex ratio for larger size classes^5^, whereas in juvenile lobsters this approaches a 1:1 ratio^4,51,53^. An effect of sex-selective harvest in which reproductive females are protected would likely lead to female skewed population patterns in legal sized lobsters, which we do not see in our model output for size classes two and three (Fig. 5). But our results do indicate that after increasing over the first three size classes, the probability of catching male lobsters levels off in the largest size class (> 101 mm CL, Fig. 5). The removal of larger males due to fisheries could be an explanation for this trend. While no female dominated population was predicted in larger lobsters—a trend that is hypothesized for fished crustacean populations due to higher fisheries mortality of larger males^6,12,13,54^—a risk of reduced reproductive success due to sperm limitation in southwestern Atlantic Canada cannot be ruled out. Our models predict spatial disparities between sexes especially for large animals. If distributions of males and females don’t overlap for extended periods or during mating season this could decrease reproductive success as lobster will not be able to find suitable mates.

The size dependency of sex ratio on both bottom temperature and water depth is most likely linked to higher mobility in larger sized lobsters^24,25,55^. Adult, large lobsters are more likely to migrate towards their preferred habitats with optimal habitat conditions (e.g. temperature, salinity) because they have lower risk of predation and are more experienced^16^. Case studies in estuaries have shown that environmental preferences differ between males and females in adulthood^5,56^. Our model predictions support these findings with skewed sex ratios in larger lobsters under different environmental conditions.

Higher water temperatures have been associated with outbreaks of the emerging epizootic shell disease (ESD) which has led to increased mortality and high economic losses in affected regions^57,58^. ESD is likely caused by an imbalanced shell bacterial community that erode the lobster cuticle^59–61^. This affects females, especially ovigerous females, to a higher degree than males^62^. Ovigerous females have longer intermolt periods, in which they carry their eggs and are more susceptible to the disease^63^. In a broader sense, female skewed populations could be at higher risk to ESD, and in turn would be more affected by rising temperatures. Here, female dominated populations were predominantly estimated for colder waters, where ESD is not as prevalent^62^. However, even in unskewed lobster stocks, under warming scenarios an above-average mortality of females can impact population dynamics in affected regions.

Our trap-based data may not reflect the true sex ratios as accurately as data obtained from dive or trawl-based surveys, although it has been reported by other studies that trap and dive surveys yield similar results^4^. Ventless traps, as used in this study, retain small lobsters and may be less selective towards larger lobsters but this potential bias could not be fully quantified. Previous studies have concluded that ventless trap provide a more accurate estimate of lobster populations, also in terms of the sex ratio^64^. We could not account for the effect of different baits used during sampling due to missing data, although it is not known if or to which degree it influences sex-specific lobster catches. Some variables with potential influence on sex ratio patterns were not available for our analyses, such as bottom salinity and substrate type. Lobsters are sensitive to salinity, with males and females having different osmoregulation capacities: Females tend to avoid lower salinities because they have to spend more energy on osmoregulation than males^5,65^. In estuaries, where salinities are low, Jury et al.^5^ reported male skewed sex ratios in American lobsters which were associated with low salinities in these areas combined with warmer temperatures. It is likely that salinity gradients confounded some of the observed sex ratio patterns in the present study, and we encourage to include this variable in future investigations. Furthermore, the bottom temperature was only recorded at the time of sampling, whereas longitudinal temperature data would have been more representative of the environmental conditions. Nevertheless, due to the consistency of the dataset this study can serve as a comprehensive baseline for future research on lobster population dynamics in the Atlantic region.

## Conclusion

This study demonstrates that the sex ratio of lobsters in southwestern Nova Scotia is influenced by the sampling time, geographical region, and that the effect of environmental factors such as bottom temperature and water depth on lobster sex ratio are dependent on lobster size. Based on our data, larger males are more likely to inhabit shallow, warmer waters whereas larger females are more likely to be found in deeper and colder waters, confirming sex ratio trends that have been reported in previous studies^4,5^. It also appears that lobster fisheries affect temporal sex ratio patterns as there is a depletion of males in LFA 33 over the course of the fishing season and the two LFAs show opposing interannual sex ratio trends that cannot be solely explained by random variation. Different responses to environmental conditions of male and female lobsters as well as different sex ratio patterns in the two LFAs call for continuous monitoring of lobster population dynamics to adapt adequate fisheries management and maintain sustainable lobster fisheries.

## Supplementary Information

Supplementary Information.
