# Supplementary material for: Spatial and temporal patterns in the sex ratio of American lobsters (Homarus americanus) in southwestern Nova Scotia, Canada
Source: Sci Rep. 2021 Dec 16;11:24100. doi: 10.1038/s41598-021-03233-8 (PMC8677755; doi:10.1038/s41598-021-03233-8)
Supplement: Supplementary file 1 — Supplementary Information. [file 41598_2021_3233_MOESM1_ESM.docx]

**Supplementary material**

**Spatial and temporal patterns in the sex ratio of American lobsters (*Homarus americanus*) in southwestern Nova Scotia, Canada**

S. Koepper ^1^, C.W. Revie ^1, 2^, H. Stryhn ^1^, K.F. Clark ^3^, S. Scott-Tibbetts ^4^, K.K. Thakur ^1^

^1^ Department of Health Management, Atlantic Veterinary College, University of Prince Edward Island, Charlottetown, C1A 4P3 PE, Canada

^2^ Department of Computer and Information Sciences, University of Strathclyde, Glasgow G1 1XQ, UK

^3^ Department of Animal Sciences and Aquaculture, Faculty of Agriculture, Dalhousie University, Truro, Nova Scotia B2N 5E3, Canada

**
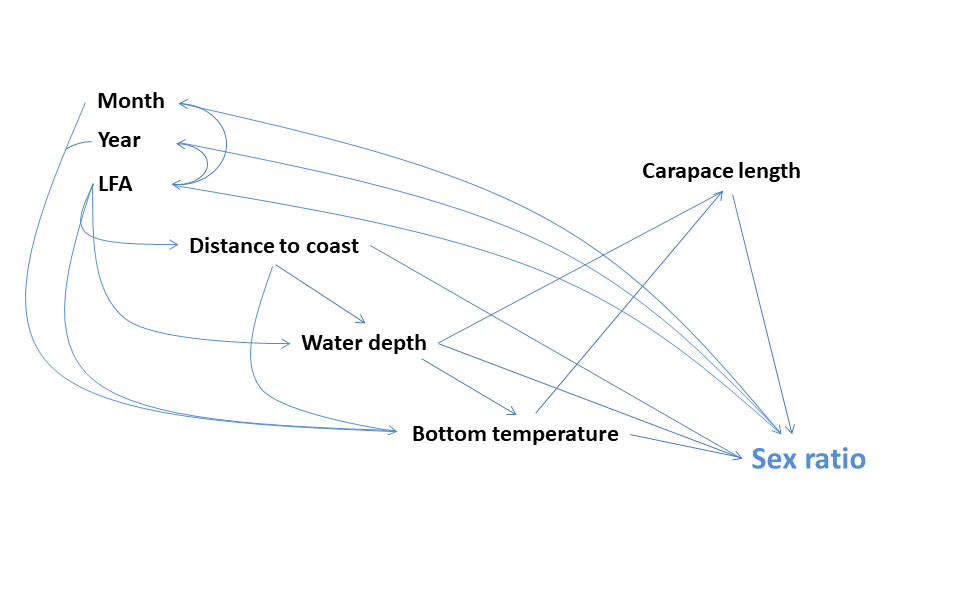
**^4^ Fishermen and Scientists Research Society, Halifax, B3M 4H4 NS, Canada

**Fig. S1:** Causal diagram displaying the effects of factors on the sex ratio patterns in lobsters.

**Tab. S1**: Model parameters for mixed effect logistic regressions including only two-way interactions (Model 1), including one three-way interaction between depth-temperature-size (Model 2), including one three-way interaction between month-year-LFA (Model 3), and including two three-way interactions between depth-temperature-size month-year-LFA (Model 4). No. IA*_i_* = number of interaction terms in model *i*; log(*L_i_*) = natural logarithm of the maximum pseudolikelihood of model *_i_*; AIC*_i_* = Akaike information criterion, Δ*_i_*AIC = difference in AIC to model with min(AIC); BIC*_i_* = Bayesian information criterion; Δ*_i_*BIC = difference in BIC to model with min(BIC). N = 249,162.

|  | No. IA*_i_* | log(*L_i_*) | AIC*_i_* | Δ*_i_*AIC | BIC*_i_* | Δ*_i_*BIC |
| --- | --- | --- | --- | --- | --- | --- |
| Model 1 | 4 | -171,868.8 | 343,823.6 | 1,012.6 | 344,271.9 | 0.0 |
| Model 2 | 3 | -171,858.5 | 342,811.0 | 0.0 | 344,301.0 | 29.1 |
| Model 3 | 3 | -171,805.5 | 343,849.0 | 1,038.0 | 345,089.7 | 817.8 |
| Model 4 | 2 | -171,794.71 | 343,835.4 | 1,024.4 | 345,117.8 | 845.9 |

**Tab. S2**: Number of sampled lobsters (and the proportion of males) by sample year and LFA collected by the Fishermen and Scientists Research Society from 2010 to 2019 in southwestern Nova Scotia, Canada (calculated on sampling event level).

|  | LFA |  |  |
| --- | --- | --- | --- |
| Year | 33 | 34 | Total |
| 2010 | 8,358 (0.53) | 6,118 (0.54) | 14,476 (0.53) |
| 2011 | 20,537 (0.53) | 17,642 (0.53) | 38,179 (0.53) |
| 2012 | 21,145 (0.54) | 18,975 (0.52) | 40,120 (0.53) |
| 2013 | 10,325 (0.53) | 9,703 (0.50) | 20,028 (0.52) |
| 2014 | 10,280 (0.54) | 7,081 (0.52) | 17,361 (0.53) |
| 2015 | 19,601 (0.53) | 11,378 (0.52) | 30,979 (0.53) |
| 2016 | 21,781 (0.53) | 14,033 (0.50) | 35,814 (0.52) |
| 2017 | 19,755 (0.50) | 12,426 (0.51) | 32,181 (0.51) |
| 2018 | 16,934 (0.52) | 10,771 (0.50) | 27,705 (0.51) |
| 2019 | 10,336 (0.52) | 5,394 (0.50) | 15,730 (0.51) |
| Total | 159,052 (0.53) | 113,521 (0.51) | **272,573 (0.52)** |
